# Effects of long noncoding RNA on prognosis of oral squamous cell carcinoma: A protocol for systematic review and meta analysis

**DOI:** 10.1097/MD.0000000000025507

**Published:** 2021-04-23

**Authors:** Qingjie Lin, Yong Zhang, Yanguo Liu, Xin Xu

**Affiliations:** a Department of Implantology, School and Hospital of Stomatology, Cheeloo College of Medicine, Shandong University & Shandong Key Laboratory of Oral Tissue Regeneration & Shandong Engineering Laboratory for Dental Materials and Oral Tissue Regeneration, Shandong University, Jinan; b Department of Implantology, Binzhou Central Hospital, Binzhou; c Department of Implantology, Jinan City People's Hospital, Jinan, Shandong Province, China.

**Keywords:** long noncoding RNA, meta-analysis, oral squamous cell carcinoma, prognosis, protocol

## Abstract

**Background:**

Long noncoding RNA (lncRNA) is reported to be upregulated in many tumors. Although the expression of lncRNA in oral squamous cell carcinoma has been assessed, the association between lncRNA expression and prognosis or clinicopathological feature still remains controversial. Therefore, we conducted a meta-analysis to verify whether lncRNA expression was related to prognosis or clinicopathological features in patients with oral squamous cell carcinoma.

**Methods:**

We searched Embase, PubMed, Web of Science, Cochrane library, Chinese National Knowledge Infrastructure, and Wanfang databases from inception to February 2021. The language included Chinese and English. The published literature on lncRNA expression and prognosis or clinicopathological characteristics of patients with oral squamous cell carcinoma was statistically analyzed. The combination of hazard ratios (HRs), odds ratios (OR), and 95% confidence intervals (95% CIs) were applied to evaluate the effects of lncRNA on the prognosis and clinicopathological features of oral squamous cell carcinoma.

**Results:**

This study could provide a comprehensive review of the available evidence of lncRNA on the prognosis and clinicopathological features of oral squamous cell carcinoma.

**Conclusion:**

The conclusion of our study will provide the updated evidence to judge the lncRNA on the prognosis and clinicopathological features of oral squamous cell carcinoma.

## 1 Introduction

Oral cancer is one of the common oral malignant tumors, accounting for more than 90%, while oral squamous cell carcinoma occupies over 80% of head and neck malignant tumors, often occurring in tongue, gingiva, buccal mucosa, and other parts of body.^[1–3]^ In recent years, the incidence of oral squamous cell carcinoma has increased year by year and the patients tended to be younger.^[4]^ Clinically, surgical treatment, radiotherapy, and chemotherapy are the main treatment methods.^[5]^ However, due to its high degree of malignancy and local invasion and lymph node metastasis, up to 60% of patients with oral squamous cell carcinoma are in the late stage of clinical progress. As a result, the survival rate of patients is low, and the life quality of patients is seriously affected.^[6]^ In the past 30 years, the overall survival (OS) rate of patients with oral squamous cell carcinoma reached about 50%, without any significant improvement.^[7]^ Early diagnosis and treatment of oral squamous cell carcinoma are the key steps to control the disease and improve the survival rate. Therefore, it is clinically important find new markers for diagnosis, prognosis, and treatment of oral squamous cell carcinoma.

With the length of more than 200bplong-stranded noncoding RNA (lncRNA) lacks the potential of protein coding and is a RNA transcription product. It is involved in DNA replication, RNA transcription, protein translation, cell development, and differentiation, and is an important regulator of cell biology.^[8–11]^ In recent years, it has been obvious that lncRNA plays an important role in the occurrence and development of tumors.^[12]^ In oral squamous cell carcinoma, the abnormal expression of lncRNA is also closely related to its occurrence and progression, and may be applied as a biomarker or therapeutic target for the diagnosis and prognosis of oral squamous cell carcinoma.^[13–16]^

Many studies have revealed that lncRNA is closely associated with the clinicopathological features, survival, and biological behavior of tumor cells in patients with oral squamous cell carcinoma.^[7,17–23]^ However, there still exists the lack of effective evidence-based medicine to prove it. Therefore, this study conducted a systematic evaluation and meta-analysis of the included articles to further explore the relationship between lncRNA and clinicopathological features and the prognosis of patients with oral squamous cell carcinoma.

## 2 Materials and methods

### 2.1 Study registration

This protocol has been registered at Open Science Framework and its registration number is DOI 10.17605/OSF.IO/Z5Q3K. Any significant amendments of this protocol will be recorded in the Open Science Framework before the review is completed. According to the Preferred Reporting Items for Systematic Reviews and Meta-analysis Protocols (PRISMA-P) statement,^[24]^ this protocol is drafted.

### 2.2 Inclusion and exclusion criteria

The study would be included in this meta-analysis if it meets the following criteria: (1) Patients were diagnosed with oral squamous cell carcinoma; (2) lncRNA expression level was detected; (3) Patients were divided into two groups based on the lncRNA expression level; (4) Efficient data were provided; (5) Full-text was available; (6) The research data were complete, and literatures on hazard ratio (HR), 95% confidence interval (CI), and observation indexes can be extracted. The following studies would be excluded from this meta-analysis: duplicated publications or patients, reviews, case reports, letters, comments, animal experiments, cell experiments, or studies without efficient data.

### 2.3 Search strategy

Embase, PubMed, Web of Science, Cochrane library, Chinese National Knowledge Infrastructure, and Wanfang databases were comprehensively searched up to February 2021. The language was Chinese and English. We also checked the references of retrieved articles to avoid missing relative studies. The combined method of MeSH Term and free words would be adopted for literature retrieval. A search strategy of PubMed is summarized in Table 1, which is created on the basis of the Cochrane handbook guidelines. The search strategies of other databases would be established similarly.

**Table 1:** Search strategy in PubMed database.

Number	Search terms
#1	Mouth Neoplasms[MeSH]
#2	Cancer of Mouth[Title/Abstract]
#3	Mouth Cancer[Title/Abstract]
#4	Oral Cancer[Title/Abstract]
#5	Oral Neoplasms[Title/Abstract]
#6	Cancer of the Mouth[Title/Abstract]
#7	Neoplasms, Mouth[Title/Abstract]
#8	Neoplasms, Oral[Title/Abstract]
#9	Cancer, Mouth[Title/Abstract]
#10	Cancer, Oral[Title/Abstract]
#11	Cancers, Mouth[Title/Abstract]
#12	Cancers, Oral[Title/Abstract]
#13	Mouth Cancers[Title/Abstract]
#14	Mouth Neoplasm[Title/Abstract]
#15	Neoplasm, Mouth[Title/Abstract]
#16	Neoplasm, Oral[Title/Abstract]
#17	Oral Cancers[Title/Abstract]
#18	Oral Neoplasm[Title/Abstract]
#19	Oral squamous cell carcinoma[Title/Abstract]
#20	or/1-19
#21	Long non-coding RNA[Title/Abstract]
#22	LncRNA[Title/Abstract]
#23	or/21-22
#24	Prognos∗
#25	Survival
#26	or/24-25
#27	#20 and #23 and #26

### 2.4 Data collection and analysis

#### 2.4.1 Selection of studies

**Figure 1:**
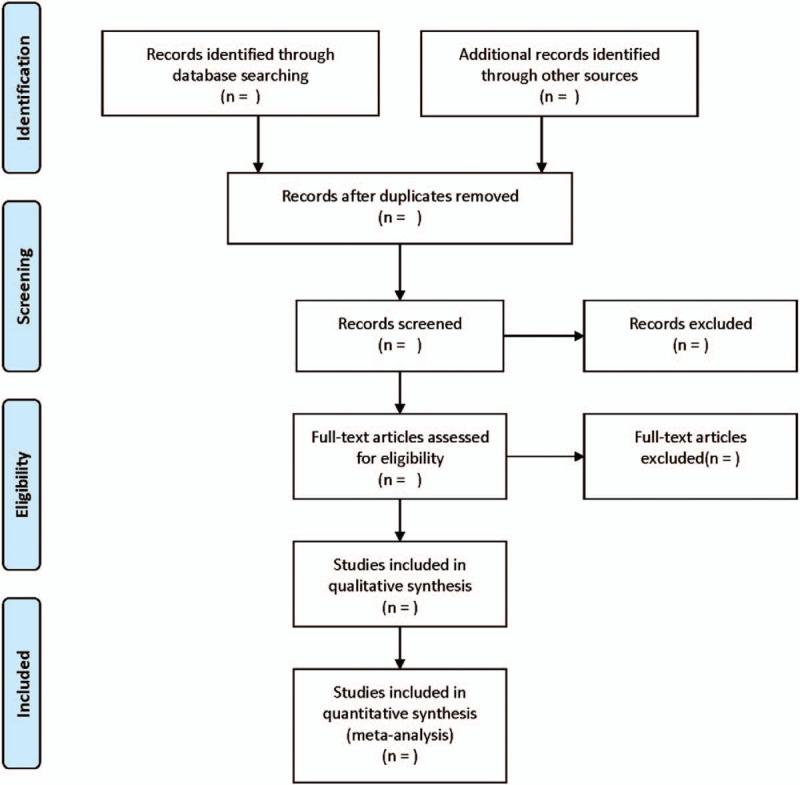
Flow diagram of study selection process.

All reviewers received evidence-based training and adhered to the summarized process. The 2 reviewers independently screened the literature based on the title, abstract, and key words of literatures, and excluded the irrelevant literatures. The rest of literatures were further confirmed by 2 researchers after reading the full text. The excluded research and the reasons for the exclusion were recorded. The differences between the 2 reviewers were resolved through consensus or a third independent reviewers. The process of the selection is illustrated in Figure 1.

#### 2.4.2 Data extraction and management

Two authors extracted the data and assessed the quality of included studies independently. Any disagreement during this process was resolved through group discussion.

According to the inclusion and exclusion criteria, the literature was screened and the relevant data were extracted. The data extracted from the literature include the first author, publication year, number of patients and region, lncRNA name, cut-off value of lncRNA expression level, detection methods, prognostic indicators such as OS and DFS, and clinicopathological data, including age, sex, tumor differentiation, tumor diameter, depth of tumor invasion, lymph node metastasis, distant metastasis, TNM stage.

#### 2.4.3 Assessment of risk of bias in included studies

The quality of all the included studies will be evaluated by 2 reviewers independently based on the Newcastle--Ottawa scale (NOS) that is used to evaluate the quality of observational studies.^[25]^ Disagreement will be reported and resolved by a third reviewer. Meanwhile, score < 6 is considered low quality, and ≥6 is classified as high quality.

#### 2.4.4 Measures of prognosis

OS and disease-free survival (DFS) would be taken as prognostic outcomes. The results would be expressed as HRs with 95% CIs.

#### 2.4.5 Dealing with missing data

If there are insufficient or missing data in the literature, we would contact the author via email. If the data is not available, we would only analyze the currently available data and discuss its potential impacts.

#### 2.4.6 Assessment of heterogeneity and data synthesis

All analysis was conducted with Review Manager 5.3 (The Cochrane Collaboration, Copenhagen, Denmark) and Stata 12.0 (Stata, College Station, TX) for Windows. For OS and DFS, HR and corresponding 95% CI were applied as the summary measures. For clinicopathological parameters, odds ratio (OR) and corresponding 95% CI were applied. Besides, inter-study heterogeneity was assessed by carrying out Chi-squared test and *I*^2^ statistic. *I*^2^ ≤ 50% or *P* value for heterogeneity >.10 proved that there was no obvious heterogeneity among studies. As a result, a fixed-effect model should be utilized. If not, a random-effect model should be applied. The association was considered to be significant when *P* < .05.

#### 2.4.7 Assessment of reporting biases

If sufficient studies are included (at least 10), we will test the reporting bias in the meta-analysis by using an inverted funnel plot.^[26]^ Funnel plots, Begg test, and Egger test were performed to evaluate the publication bias.

#### 2.4.8 Subgroup analysis

We will conduct a subgroup analysis based on the detection method of lncRNA expression, race, and the source of survival data.

#### 2.4.9 Sensitivity analysis

If sufficient studies are available, we will perform sensitivity analyses to confirm the robustness of the primary results. The meta-analysis will be respectively processed by excluding studies with small sample size, and low methodological quality.

#### 2.4.10 Ethics and dissemination

The content of this article does not involve moral approval or ethical review and would be presented in print or at relevant conferences.

## 3 Discussion

A large number of studies at home and abroad have confirmed that lncRNA is involved in the pathological process of the occurrence and development of a variety of tumors at different levels.^[27–29]^ lncRNA can be used as a regulator of gene expression through gene modification, transcription, and post-transcriptional processing. Many studies have revealed that lncRNA can participate in the proliferation, apoptosis, angiogenesis, metastasis, and invasion of oral squamous cell carcinoma through various pathways and molecular mechanisms.^[30–32]^ In the past few years, a large number of studies have illustrated that the upregulation and downregulation of lncRNA are involved in the development and progression of oral squamous cell carcinoma.^[33,34]^ Therefore, the expression of lncRNA may be related to the prognosis of oral squamous cell carcinoma, while controversial results have been discovered. Here, we conducted this meta-analysis for the first time to further summarize the prognostic value of lncRNA expression in oral squamous cell carcinoma.

There are several key points in our research. First of all, as far as we know, this is the first meta-analysis to explore the prognostic value of lncRNA expression in oral squamous cell carcinoma. Second, this study comprehensively analyzed the prognostic and pathological parameters, which further confirmed the prognostic role of lncRNA expression in oral squamous cell carcinoma. Third, our research strictly follows the rules of PRISMA, so the method is normative. Nevertheless, our research has no limitations. There are few studies on the meta-analysis, and a relatively small sample size may reduce the reliability of the results. Furthermore, there is a great difference in the critical value among the included studies, which may limit the clinical application of this conclusion. Most importantly, although there are no restrictions on the country in the process of literature selection, most of the studies are carried out in China. Consequently, this conclusion may be difficult to extend to other countries. Therefore, more high-quality and extensive researches should be carried out to clarify this issue.

Nevertheless, our study will provide evidence to support the prognostic role of lncRNA expression in oral squamous cell carcinoma and provide relevant strategies for the accurate treatment of tumors.

## Author contributions

**Data curation:** Qingjie Lin and Yong Zhang.

**Formal analysis:** Yong Zhang.

**Methodology:** Yong Zhang.

**Project administration:** Yong Zhang.

**Supervision:** Yanguo Liu.

**Validation:** Yanguo Liu.

**Visualization and software:** Yanguo Liu.

**Writing – original draft:** Qingjie Lin and Xin Xu.

**Writing – review & editing:** Qingjie Lin and Xin Xu.

**Conceptualization:** Xin Xu, Qingjie Lin.

**Data curation:** Qingjie Lin, Yong Zhang.

**Funding acquisition:** Xin Xu.

**Investigation:** Yong Zhang.

**Methodology:** Yong Zhang.

**Project administration:** Xin Xu.

**Resources:** Yong Zhang.

**Supervision:** Yanguo Liu.

**Validation:** Yanguo Liu.

**Visualization:** Yanguo Liu.

**Writing – original draft:** Xin Xu, Qingjie Lin.

**Writing – review & editing:** Xin Xu, Qingjie Lin.
